# Exploratory study on the ascending pain pathway in patients with chronic neck and shoulder pain based on combined brain and spinal cord diffusion tensor imaging

**DOI:** 10.3389/fnins.2025.1460881

**Published:** 2025-02-12

**Authors:** Zhiqiang Qiu, Tianci Liu, Chengxi Zeng, Maojiang Yang, HongYing Yang, Xiaoxue Xu

**Affiliations:** ^1^Department of Radiology, Affiliated Hospital of North Sichuan Medical College, Nanchong, China; ^2^Department of Pain, Affiliated Hospital of North Sichuan Medical College, Nanchong, China

**Keywords:** chronic neck and shoulder pain, ascending pain pathway, combined imaging of brain and spinal cord, diffusion tensor imaging, DTI

## Abstract

**Objective:**

To explore the changes in the white matter microstructure of the ascending pain conduction pathways in patients with chronic neck and shoulder pain (CNSP) using combined brain and spinal cord diffusion tensor imaging techniques, and to assess its correlation with clinical indicators and cognitive functions.

**Materials and methods:**

A 3.0T MRI scanner was used to perform combined brain and spinal cord diffusion tensor imaging scans on 31 CNSP patients and 24 healthy controls (HCs), extracting the spinothalamic tract (STT) and quantitatively analyzing the fractional anisotropy (FA) and mean diffusivity (MD) which reflect the microstructural integrity of nerve fibers. Additionally, these differences were subjected to partial correlation analysis in relation to Visual Analog Scale (VAS) scores, duration of pain, Self-Rating Anxiety Scale (SAS), and Self-Rating Depression Scale (SDS).

**Results:**

Compared to HCs, CNSP patients showed decreased mean FA values and increased mean MD values in bilateral intracranial STT compared to the HC group, but two-sample *t*-test results indicated no statistically significant differences (*p* > 0.05). FA values of the left STT (C2 segment, C5 segment) and right STT (C1 segment, C2 segment) were significantly decreased in bilateral cervical STTs of CNSP patients; MD values of the left STT (C1 segment, C2 segment, C5 segment) and right STT (C1 segment, C5 segment) were significantly increased (*p* < 0.05). Partial correlation analysis results showed that FA values of STT in CNSP patients were negatively correlated with VAS scores, duration of pain, SAS scores, and SDS scores, while MD values were positively correlated with VAS scores and duration of pain (Bonferroni *p* < 0.05).

**Conclusion:**

This research identified that patients with CNSP exhibited reduced mean FA and increased mean MD in the bilateral intracranial STT, although these differences were not statistically significant (*p* > 0.05). Conversely, significant abnormalities were observed in specific segments of the bilateral cervical STT (*p* < 0.05), which were also correlated with variations in pain intensity, illness duration, and levels of anxiety and depression. These findings contribute a novel neuroimaging perspective to the evaluation and elucidation of the pathophysiological mechanisms underlying chronic pain in the ascending conduction pathways.

## 1 Introduction

Chronic Neck and Shoulder Pain (CNSP) is one of the most common symptoms of cervical spine disease, defined as pain in the neck and shoulder region lasting more than 3 months (Cohen, 2015). Globally, the annual incidence rate of CNSP is estimated to be between 10.4 and 21.3% (Hoy et al., 2010). Besides causing long-term pain, CNSP can also interfere with a patient’s daily attention, emotional state, and cognitive functions, severely affecting their quality of life (Hofbauer et al., 2001).

Clinically, the treatment of CNSP primarily involves conservative therapies, such as medication, exercise, and physical therapy. For patients with more severe symptoms, surgical treatment may be necessary. Despite this, many patients suffer from long-term pain due to poor treatment outcomes, and it has been reported that CNSP has become the fourth leading cause of disability worldwide (Bakhsheshian et al., 2017). Given this, it is imperative to develop more effective treatment strategies. Recent studies indicate that interventions targeting the ascending pain pathways have shown significant potential (Shi and Wu, 2023). However, the specific abnormal mechanisms of the ascending pain conduction pathways in CNSP patients are not yet fully understood.

Moreover, current research primarily focuses on exploring the functional and structural impacts of chronic pain on the brain (Malfliet et al., 2017). However, the central nervous system is composed of both the brain and spinal cord, and the mechanisms of pain effects in the ascending and descending pathways between the spinal cord and brain are not yet clear (Baron et al., 2010).

The combined imaging technology of the brain and spinal cord can simultaneously obtain neurobiological signals from both, facilitating the observation of correlations between lower and higher central nervous systems and allowing a holistic study of the central nervous system’s conduction mechanisms, which holds tremendous application prospects (Tinnermann et al., 2021).

Nociceptive sensory information from peripheral sensory organs (including intensity, location, and nature) are conveyed to the thalamus via the spinothalamic tract (STT), where it undergoes modulation (Willis and Westlund, 1997). This processed information is subsequently relayed to the primary (S1) and secondary (S2) somatosensory cortices for further encoding (Coghill et al., 1999). Information related to the nociceptive affective component is transmitted via the spinoreticular tract to the brainstem’s reticular formation, further sent to the thalamus, where it is integrated and projected to cortical and subcortical structures (Hunt and Mantyh, 2001). The cognitive modulation of pain processing is primarily mediated by frontal cortex regions, including the anterior cingulate cortex, ventromedial prefrontal cortex, and dorsolateral prefrontal cortex (Lui et al., 2010). Brain regions classically associated with the affective dimension of pain, such as the secondary somatosensory cortex and anterior insular cortex, are integral to the perception of pain’s unpleasantness (Schreckenberger et al., 2005).

Recent neuroimaging studies have revealed that CNSP patients exhibit significant cortical thinning and abnormalities in brain metabolic function in the S1 area (Woodworth et al., 2019). Furthermore, these patients also show reduced functional connectivity between the thalamus and the S1 region (Yang et al., 2020). Sprenger et al. (2015). combines spinal cord and cortical fMRI to explore the relationship between spinal cord-midbrain connectivity and subjective pain intensity, It was found that when the subjects received thermal stimulation on the forearm, the functional connectivity between the C6-dorsal horn and the thalamus, SI, the bilateral insula, the bilateral striatum, the bilateral amygdala, the hypothalamus, and the midbrain was enhanced, and the strength of the functional connectivity was positively correlated with the average pain score. Studies also suggest that in chronic pain states, neural pathways may undergo central sensitization, an adaptive change in the central nervous system that leads to the amplification and spread of nociceptive signals, causing an increase in the persistence and intensity of pain (Ji et al., 2018). Therefore, we speculate that the white matter microstructure of the STT, which transmits these nociceptive information, may be damaged in CNSP patients.

In order to verify our hypothesis, this study will employ combined brain and spinal cord diffusion tensor imaging (DTI) techniques to explore the plastic changes in the pain transmission pathways in CNSP. Additionally, this study also collects clinical and cognitive function data including pain intensity, duration of illness, and levels of anxiety and depression, and conducts partial correlation analysis with changes in STT white matter microstructure. This will provide new neuroimaging evidence for the evaluation and understanding of the pathophysiological basis of such chronic pain in the ascending conduction process, and may also facilitate the development of intervention strategies for pain transmission pathways, such as neuromodulation therapies targeting specific brain regions or spinal segments (Knotkova et al., 2021). Additionally, this non-invasive method of detecting damage in the pain transmission pathways of patients helps promote personalized pain management strategies.

## 2 Methods

All research methodologies were sanctioned by the Ethics Committee of the North Sichuan Medical College Affiliated Hospital, adhering strictly to the guidelines outlined in the Declaration of Helsinki. The ethical approval for this study was assigned the number 2023ER95-1. Prior to participation, researchers provided a comprehensive explanation of the study’s objectives and procedures to the participants, and all participants provided written informed consent.

### 2.1 Participants

#### 2.1.1 CNSP group

Participants were diagnosed with chronic pain by two experienced pain specialists at the Affiliated Hospital of North Sichuan Medical College, following the chronic pain classification criteria outlined in the International Classification of Diseases, 11th Revision (ICD-11; Scholz et al., 2019). Inclusion Criteria:(1) Persistent neck and shoulder pain, with or without radiating arm pain(unilateral or bilateral), lasting at least 3 months, corroborated by radiographic evidence of cervical degeneration on X-ray or MRI;(2) Routine MRI examination showed no morphological changes in the spinal cord (ruling out conditions such as cervical spondylotic myelopathy that may cause morphological changes in the spinal cord);(3) Age between 20 and 70 years, right-handed;(4) No contraindications to MRI; (5) Absence of significant pain in other body regions. Exclusion Criteria: (1) Gross abnormalities, such as infarction, hemorrhage, or brain tumors. The Fazekas score (range, 0–3), was either 0 (indicating absence of abnormalities) or 1 (indicating the presence of caps, pencil-thin linings, and/or punctate foci) (Inano et al., 2011); (2) Primary psychiatric disorders including anxiety, depression, Alzheimer’s disease, schizophrenia, or other neurologic or psychiatric disorders;(3) Inability to tolerate prolonged MRI scans due to severe pain;(4) Severe underlying cardiac, hepatic, or renal diseases.

#### 2.1.2 HCs group

Inclusion criteria: (1) Age and handedness matched with the CNSP group; (2) No MRI contraindications; (3) No acute or chronic pain symptoms. Exclusion criteria: (1) Gross abnormalities, such as infarction, hemorrhage, or brain tumors. The Fazekas score (range, 0–3), was either 0 (absence) or 1 (caps, pencil-thin linings, and/or punctate foci); (2) Neurological or psychiatric disorders.

### 2.2 Psychological assessment

All assessments were conducted before the MRI scan through face-to-face interviews and completion of questionnaires, using the Visual Analog Scale (VAS) to evaluate the average pain intensity of the subject over the past week, ranging from 0 (no pain) to 10 (the worst imaginable pain). Concurrently, levels of anxiety and depression were measured using the Self-Rating Anxiety Scale (SAS) (Zung, 1965) and the Self-Rating Depression Scale (SDS) (Zung, 1971), respectively. The duration of pain is defined as the time between the initial CNSP diagnosis date and the preoperative brain MRI acquisition date.

### 2.3 Imaging acquisition

All MRI scans were performed using a Siemens MAGNETOM Skyra 3.0 T MRI scanner and a standard 20-channel head neck combined coil. Subjects lay supine on the examination bed, with their heads comfortably positioned and secured with foam to minimize movement, earplugs were worn to reduce external noise, and they were instructed to stay awake, close their eyes, and keep their heads still. All imaging data were collected in a single scan (only one localization scan was performed), first acquiring T1 high-resolution structural image and diffusion magnetic resonance imaging (dMRI) data of the brain, followed by the acquisition of T1 high-resolution structural image and dMRI data of the spinal cord using another field of view (FOV). The total time for completing all imaging data scans was 27 min and 52 s.

#### 2.3.1 Brain image

The T1 high-resolution structural image is acquired using a three-dimensional (3D) Magnetization Prepared Rapid Gradient Echo (MP-RAGE) sequence with the following parameters: repetition time (TR) = 2,240 ms; inversion time (TI) =1,130 ms; data matrix =256 × 256; FOV =256 × 256 mm; scanning orientation is sagittal; slices = 192; slice thickness = 1 mm without gap; voxel size = 1.0 × 1.0 × 1.0 mm; scan time = 4 min and 46 s.

The dMRI data is acquired at 2 mm isotropic resolution using an echo-planar imaging (EPI) sequence with the following parameters: multiband factor (MB) = 4; 30 noncoplanar diffusion directions with b = 1,000 s/mm^2^; 5 AP (Anterior-to-Posterior) and 5 PA (Posterior-to-Anterior) images with b = 0 s/mm^2^; TR = 10,500 ms; echo time (TE) = 92 ms; data matrix = 128 × 128; FOV = 256 mm × 256 mm; scanning orientation is transverse; slices = 72; slice thickness = 2 mm without gap; voxel size = 2.0 × 2.0 × 2.0 mm; scan time = 7 min and 59 s.

#### 2.3.2 Cervical spinal cord image

The T1 high-resolution structural image is acquired using a three-dimensional (3D) Magnetization Prepared Rapid Gradient Echo (MP-RAGE) sequence with the following parameters: repetition time (TR) = 2,080 ms; inversion time (TI) =1,050 ms; data matrix =256 × 128; field of view (FOV) =256 × 128 mm; scanning orientation is sagittal; slices = 192; slice thickness = 1 mm without gap; voxel size = 1.0 × 1.0 × 1.0 mm; scan time = 4 min and 1 s.

The diffusion magnetic resonance imaging (dMRI) data is acquired using an echo-planar imaging (EPI) sequence with the following parameters: multiband factor (MB) = 4; 30 noncoplanar diffusion directions with b = 800 s/mm^2^; 5 AP (Anterior-to-Posterior) and 5 PA (Posterior-to-Anterior) images with b = 0 s/mm^2^; TR = 4,050 ms; echo time (TE) = 86 ms; data matrix = 192 × 96; FOV = 250 mm × 125 mm, to eliminate phase-wrap in the phase direction, we add 80% oversampling during acquisition; scanning orientation is sagittal; slices = 20; slice thickness = 3 mm without gap; voxel size = 1.3 × 1.3 × 3.0 mm; scan time = 11 min and 6 s.

### 2.4 Brain image data preprocessing

The preprocessing of diffusion MRI data is a critical step for the accurate computation of diffusion tensors and reliable tractography. The precision of these preprocessing techniques is directly linked to the accuracy of fiber pathway reconstruction and the quantitative analysis of neural tracts. To ensure data integrity and analytical accuracy, this study employed a series of rigorous preprocessing protocols. First, data quality was carefully assessed by evaluating resolution, gradient direction counts, b-values, signal-to-noise ratios, artifacts, and head motion parameters. Following this, the MRI data were converted from DICOM to NIFTI format using the dcm2niix tool to enable further processing. Given the spatial dependency of noise in diffusion data collected from multi-channel receiver coils (Aja-Fernández et al., 2014; André et al., 2014), thermal noise was corrected using the Marchenko–Pastur Principal Component Analysis (MP-PCA; Veraart et al., 2016a,b) method implemented in MRtrix3. To address artifacts resulting from k-space truncation or finite sampling (Kellner et al., 2016), Gibbs ringing correction was also applied using MRtrix3. Since EPI-based diffusion data are prone to distortions caused by magnetic field inhomogeneities, EPI distortion correction was performed using the topup tool in FSL, which utilized b = 0 data with anteroposterior (AP) and posteroanterior (PA) phase encoding for accurate correction (Andersson et al., 2003). Additionally, motion-related distortions and eddy-current effects were mitigated using the eddy tool in FSL (Andersson et al., 2016). Intensity inhomogeneities caused by low-frequency intensity shifts in MR images were corrected with the N4BiasFieldCorrection algorithm (Tustison et al., 2010) available in the ANTs software. Finally, non-brain structures, such as the scalp and skull, were removed using the Brain Extraction Tool (BET) in FSL, which ensured accurate spatial registration and reduced computational burden, thereby enhancing the focus and effectiveness of fiber tracking.

### 2.5 Deterministic fiber tracking

**Figure 1:**
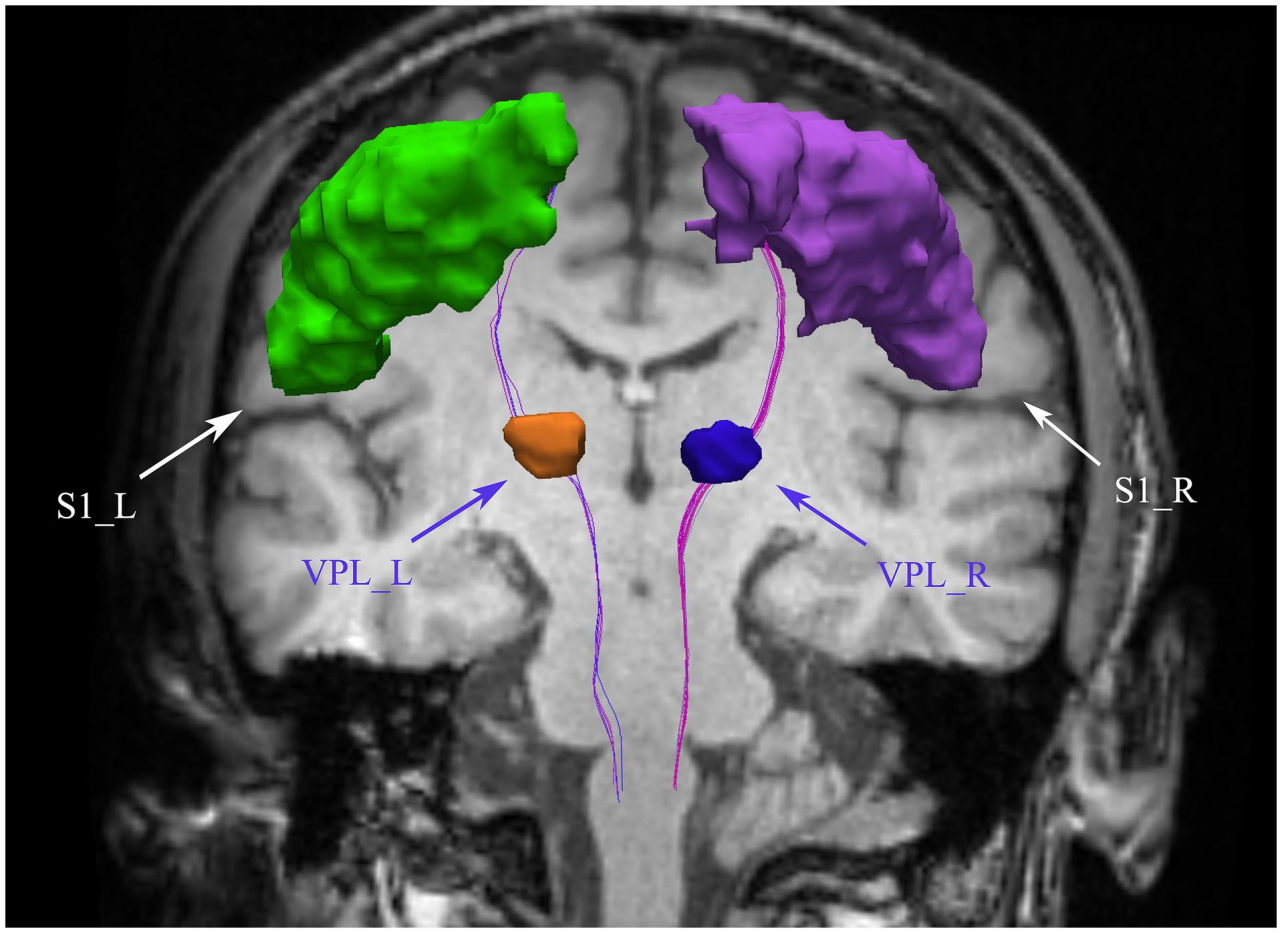
Bilateral spinothalamic tract fiber tracks. The figure demonstrates bilateral spinothalamic tract of a representative patient with CNSP. VPL, ventral posterior lateral nucleus of the thalamus; S1, primary somatosensory cortex; L, left; R, right.

Tensor estimation is performed on a voxel-by-voxel basis using the DTIFIT tool within FSL software, allowing for the calculation of key diffusion parameters such as fractional anisotropy (FA) and mean diffusivity (MD), which help characterize neural tissue. Following tensor estimation, whole-brain tractography is carried out using a streamline tracking algorithm implemented in the Diffusion Toolkit software (Ruopeng Wang, Van J. Wedeen, TrackVis.org, Martinos Center for Biomedical Imaging, Massachusetts General Hospital). The parameters for this process include a step size of 1 mm, a fractional anisotropy threshold of 0.2, and an angular threshold of 30° (Basser et al., 2000), ensuring accurate tracking of fiber pathways. Fiber tract segmentation is then conducted using the waypoint ROI procedure, as described by Wakana et al. (2007). In this approach, fiber tracts are classified based on their intersection with two predefined ROI waypoints that delineate their trajectory. The whole-brain tractography data is imported into TrackVis software, and two regions of interest (ROIs) are defined in the VPL and S1 regions to isolate the spinothalamic tract (STT), according to anatomical definitions provided by Antonio et al. (refer to Figure 1). To accurately map these ROIs from a standard atlas (Tian et al., 2020; Yeo et al., 2011) into individualized brain spaces, both linear and nonlinear registration techniques are applied using the ANTs (Avants et al., 2011) software. Finally, mean FA and MD values are calculated along the segmented STT fiber tracts. Variations in these metrics provide insights into microstructural differences within the fiber pathways, which can be used for further analysis of neural tissue integrity.

### 2.6 Atlas-based cervical spinal cord white matter tract processing

**Figure 2:**
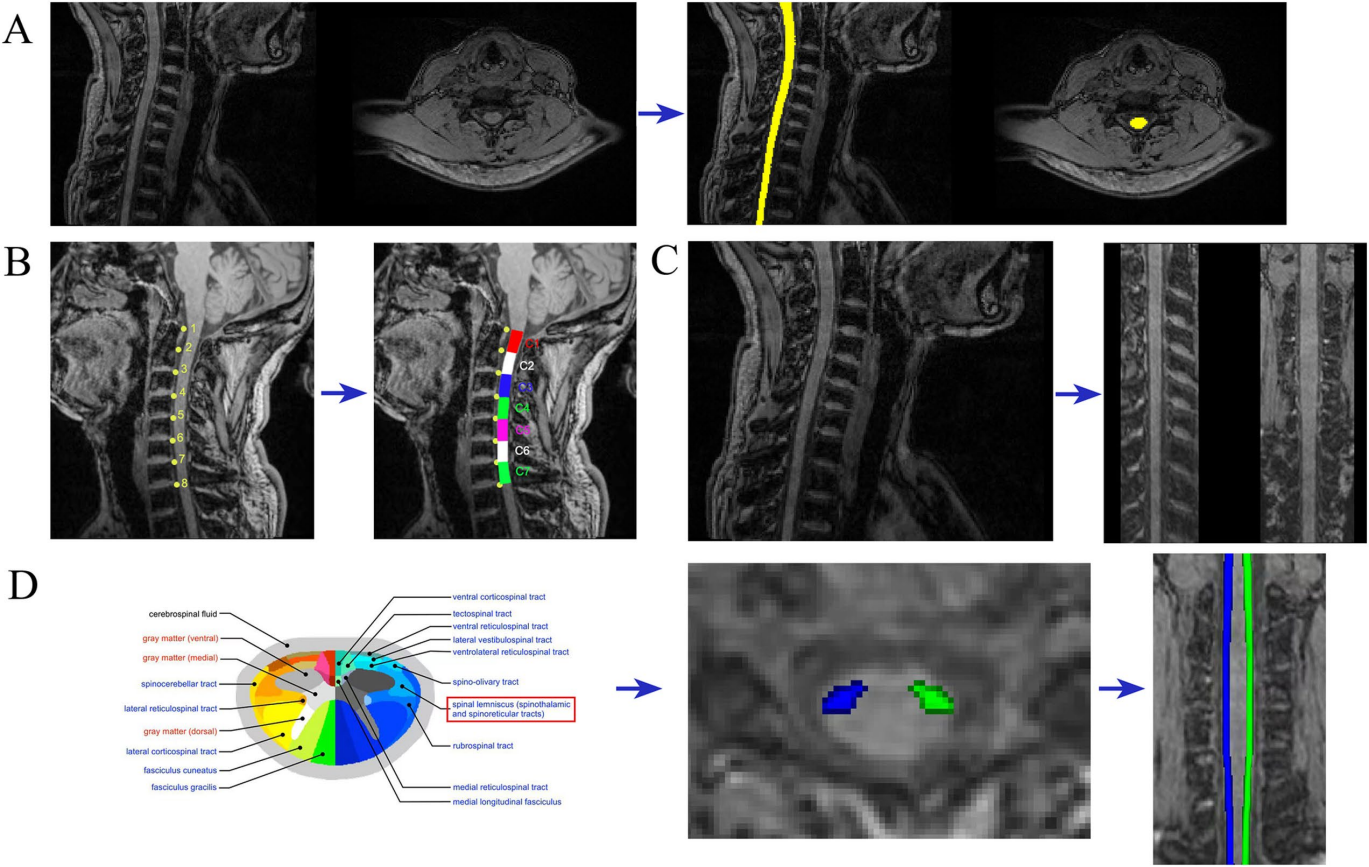
Imaging analysis. Examples of MRI metric analysis showing **(A)** spinal cord segmentation; **(B)** spinal cord segmental delineation; **(C)** spinal cord “straightened” **(D)** spinothalamic tract segmentation.

Image processing and analysis of the MRI data are carried out using the Spinal Cord Toolbox (SCT; De Leener et al., 2017a), following a structured workflow. Initially, the MRI data are converted from DICOM to NIFTI format utilizing the dcm2niix tool, preparing the data for further processing. The spinal cord is then automatically segmented using SCT’s deep learning segmentation tool (De Leener et al., 2014; Gros et al., 2018) (sct_deepseg_sc), and manual corrections are applied when necessary through FSLeyes to address any segmentation inaccuracies (refer to Figure 2A). For spinal cord segmental delineation, the segmentation is performed on T1-weighted imaging (T1WI) according to cervical vertebrae levels (Cohen-Adad et al., 2021; De Leener et al., 2015), with each segment encompassing the corresponding vertebra and its adjacent intervertebral space (refer to Figure 2B). The spinal cord is subsequently “straightened” using SCT’s registration tool, aligning it with the PAM50 template (De Leener et al., 2017b; De Leener et al., 2018) (refer to Figure 2C). This step generates a warp field that will be used in subsequent registrations. The topup tool from FSL (Andersson et al., 2003) is used to correct the susceptibility-induced off-resonance distortions by leveraging the AP/PA image pairs. Following this, the eddy tool is employed to correct for eddy current-induced distortions and subject motion (see Supplementary Figures S1, S2 for the effect images before and after correction by topup and eddy). Motion correction is further refined using the sct_dmri_moco tool within SCT. Next, the corrected DTI images are registered to the PAM50 template (De Leener et al., 2018) using the warp field obtained during the earlier registration step. With this alignment in place, the bilateral spinothalamic tracts are extracted using the spinal cord white matter template (Dupont et al., 2017) provided by SCT. The atlas was built from the Gray’s Anatomy, which illustrates the position of the 15 different WM tracts of both left and right sides at the mid-cervical level in human (Figure 2D) (Lévy et al., 2015). Finally, fiber tract quantification is performed using SCT’s function (sct_extract_metric) to calculate fractional anisotropy (FA) and mean diffusivity (MD) values for the bilateral spinothalamic tracts (STT) across the cervical segments from C1 to C7, utilizing the maximum posterior probability map approach (Nopriadi, 2012) to ensure precise quantification. This method introduces prior information, making the estimation results more robust in the context of spinal cord dMRI data, which typically has high noise and a low signal-to-noise ratio.

### 2.7 Statistical analysis

Pearson’s chi-squared test was employed to evaluate gender disparities across groups. Additionally, age differences between groups were assessed using the two-sample t-test, with statistical significance set at *p* < 0.05.

For each participant’s bilateral STT, the mean FA and MD values were computed across the core of the tract. These values were then compared between groups using the two-sample t-test, with statistical significance set at *p* < 0.05.

Independent two-sample *t*-tests were performed to compare the FA and MD values across the cervical spinal cord segments of the spinothalamic tract between the patient and control groups. FA and MD change curves were subsequently plotted, highlighting segments with statistically significant differences. To further explore the relationships among the diffusion properties of patients with CNSP, duration of pain, VAS scores, SAS scores, and SDS scores, a partial correlation analysis was conducted. Considering that age may have a significant effect on these individual behavioral measures, controlling for age as a covariate. Bonferroni correction was applied to account for multiple comparisons, with statistical significance set at Bonferroni *p* < 0.05.

## 3 Results

In this study, a cohort of 34 patients with CNSP was initially recruited. However, one patient withdrew from the MRI procedure due to pain, and two others were excluded due to excessive head motion during data quality assessment, resulting in a final sample of 31 CNSP patients. Additionally, 24 healthy controls were included in the study. No significant statistical differences were observed in age and gender between the two groups (*p* > 0.05). CNSP patients exhibited significantly elevated SAS and SDS scores compared to the HCs (*p* < 0.05). Detailed clinical and demographic data for both groups are presented in Table 1.

**Table 1:** Demographic and behavioral data.

	CNSP (*n* = 31)	HCs (*n* = 24)	*p* value
Gender (male/female)	15/16	11/13	0.851
Age (years)	49.68 ± 7.34	47.45 ± 8.52	0.286
Duration of pain (months)	38.56 ± 17.34	–	–
Pain area (left-sided pain/right-sided pain/bilateral pain)	8/9/14	–	–
VAS	6.12 ± 1.34	–	–
SAS	43.53 ± 10.34	34.76 ± 5.43	0.007
SDS	44.36 ± 12.34	36.42 ± 6.28	0.012

CNSP, chronic neck and shoulder pain; HCs, healthy controls; VAS, visual analog scale; SAS, Self-Rating Anxiety Scale; SDS, Self-Rating Depression Scale.

### 3.1 Bilateral spinothalamic tracts extract results

This study employed deterministic fiber tracking techniques to extract the bilateral intracranial spinothalamic tracts of 31 CNSP patients and 24 HCs, with ROIs being the bilateral VPL and bilateral S1 areas, as shown in Figure 1 with the extraction results of a CNSP case. Additionally, using the white matter templates provided by SCT software, the bilateral cervical spinothalamic tracts of all subjects were extracted, as shown in Figure 2D, displaying the extraction results of a CNSP case.

### 3.2 Inter-group analysis of mean FA and MD in bilateral intracranial spinothalamic tracts

In the bilateral STT, the CNSP group exhibited lower mean FA values compared to the HCs, although the differences were not statistically significant (left STT *p* = 0.165, right STT *p* = 0.243, both > 0.05) as detailed in Table 2. Similarly, the CNSP group demonstrated higher mean MD values than the HCs, but these differences also did not reach statistical significance (left STT *p* = 0.147, right STT *p* = 0.216, both > 0.05); refer to Table 2 for further data.

**Table 2:** Comparison of average diffusion index of spinothalamic tract between groups.

Diffusion metrics	CNSP	HCs	*t*	*p*
FA_L	0.531 ± 0.035	0.539 ± 0.042	−1.142	0.165
FA_R	0.538 ± 0.036	0.546 ± 0.045	−0.889	0.243
MD_L	0.778 ± 0.068	0.769 ± 0.060	1.125	0.147
MD_R	0.773 ± 0.062	0.763 ± 0.051	0.912	0.216

CNSP, chronic neck and shoulder pain; HCs, healthy controls; FA, fractional anisotropy; MD, mean diffusivity; L, left; R, right.

### 3.3 Segmental analysis of FA and MD values in bilateral cervical spinothalamic tracts between groups

**Figure 3:**
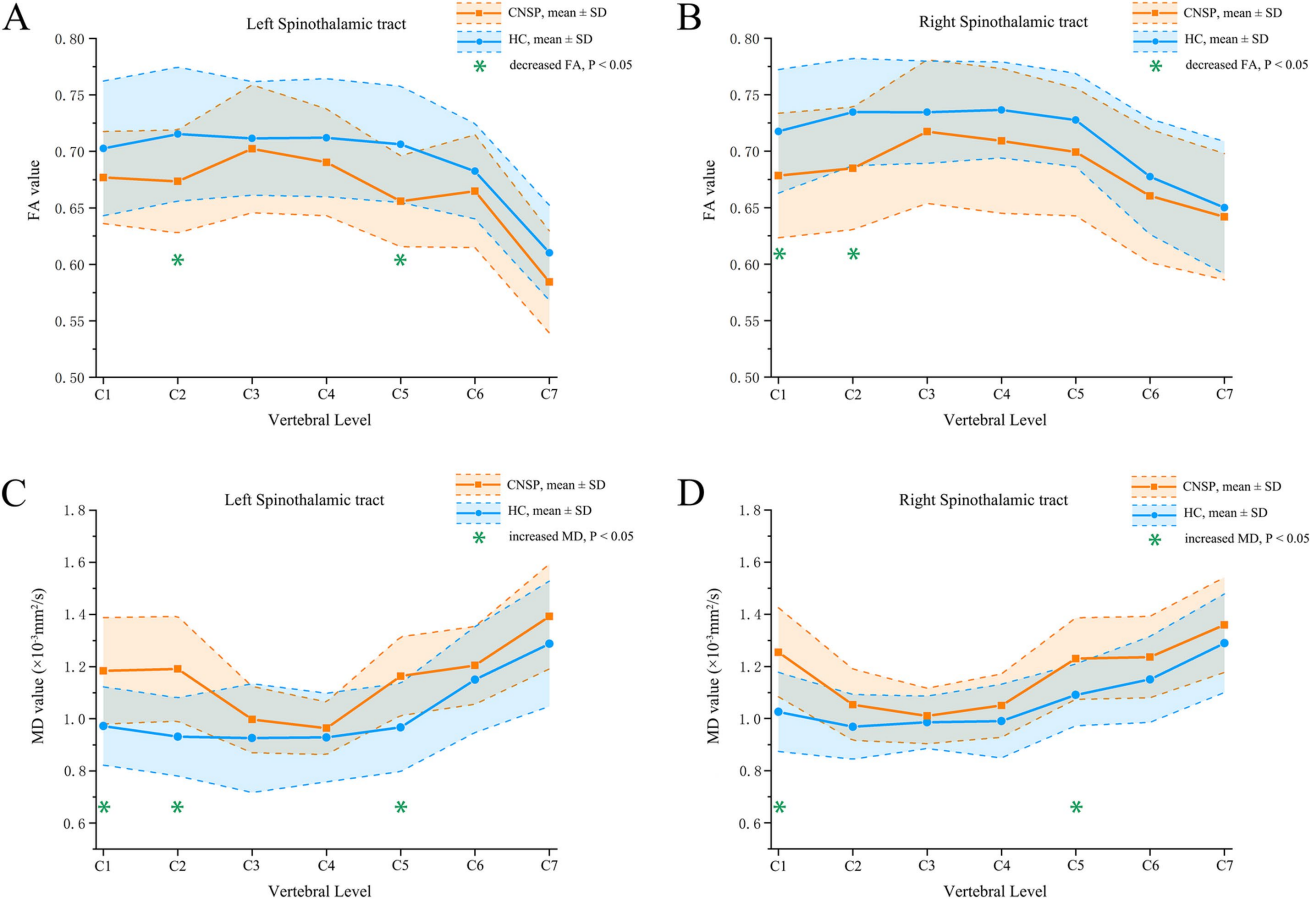
Segmental analysis of FA and MD values between groups. **(A)** inter-group comparison of FA values in the left spinothalamic tract; **(B)** inter-group comparison of FA values in the right spinothalamic tract; **(C)** inter-group comparison of MD values in the left spinothalamic tract; **(D)** inter-group comparison of MD values in the right spinothalamic tract. CNSP, chronic neck and shoulder pain; HCs, healthy controls; FA, fractional anisotropy; MD, mean difusivity; SD, standard deviation.

FA analysis: Compared to the HCs group, the CNSP group showed a significant decrease in FA values in the left STT C2 segment (*p* = 0.017), left STT C5 segment (*p* = 0.012), right STT C1 segment (*p* = 0.025) and right STT C2 segment (*p* = 0.014), refer to Table 3 and Figures 3A,B for further data.

**Table 3:** Segmental analysis of FA values in bilateral cervical spinothalamic tracts between groups.

Fiber tract	CNSP	HCs	*t*	*p*
C1_L	0.676 ± 0.039	0.702 ± 0.059	−1.308	0.215
C2_L	0.673 ± 0.045	0.715 ± 0.055	−3.176	0.017
C3_L	0.702 ± 0.056	0.711 ± 0.044	−0.576	0.534
C4_L	0.690 ± 0.047	0.712 ± 0.062	−0.942	0.254
C5_L	0.655 ± 0.038	0.706 ± 0.051	−3.754	0.012
C6_L	0.664 ± 0.049	0.682 ± 0.042	−1.268	0.285
C7_L	0.584 ± 0.042	0.610 ± 0.038	−1.457	0.226
C1_R	0.677 ± 0.047	0.610 ± 0.038	−2.853	0.025
C2_R	0.681 ± 0.045	0.734 ± 0.067	−3.546	0.014
C3_R	0.716 ± 0.054	0.733 ± 0.085	−0.856	0.354
C4_R	0.707 ± 0.044	0.736 ± 0.072	−1.026	0.245
C5_R	0.698 ± 0.049	0.727 ± 0.091	−1.564	0.198
C6_R	0.662 ± 0.052	0.677 ± 0.115	−0.856	0.235
C7_R	0.642 ± 0.041	0.649 ± 0.086	−0.542	0.612

CNSP, chronic neck and shoulder pain; HCs, healthy controls; FA, fractional anisotropy; L, left; R, right.

MD analysis: Compared to the HC group, the CNSP group showed a significant increase in MD values in the left STT C1 segment (*p* = 0.023), left STT C2 segment (*p* = 0.018), left STT C5 segment (*p* = 0.034), right STT C1 segment (*p* = 0.021) and right STT C5 segment (*p* = 0.036) refer to Table 4 and Figures 3C,D for further data.

**Table 4:** Segmental analysis of MD values in bilateral cervical Spinothalamic tracts between groups.

Fiber tract	CNSP (×10^−3^ mm^2^/s)	HCs (×10^−3^ mm^2^/s)	*t*	*p*
C1_L	1.182 ± 0.25	0.972 ± 0.19	3.503	0.023
C2_L	1.196 ± 0.21	0.930 ± 0.28	3.875	0.018
C3_L	0.997 ± 0.15	0.925 ± 0.12	0.976	0.227
C4_L	0.963 ± 0.11	0.928 ± 0.25	0.458	0.573
C5_L	1.162 ± 0.21	0.967 ± 0.16	2.426	0.034
C6_L	1.242 ± 0.19	1.152 ± 0.22	0.746	0.349
C7_L	1.397 ± 0.22	1.291 ± 0.27	1.145	0.186
C1_R	1.252 ± 0.18	1.032 ± 0.12	3.667	0.021
C2_R	1.054 ± 0.13	0.968 ± 0.24	1.126	0.194
C3_R	1.012 ± 0.16	0.985 ± 0.10	0.374	0.654
C4_R	1.057 ± 0.21	0.990 ± 0.15	0.954	0.272
C5_R	1.233 ± 0.17	1.091 ± 0.11	2.572	0.036
C6_R	1.243 ± 0.24	1.152 ± 0.20	0.941	0.198
C7_R	1.361 ± 0.26	1.294 ± 0021	1.076	0.175

CNSP, chronic neck and shoulder pain; HCs, healthy controls; MD, mean diffusivity; L, left; R, right.

### 3.4 Partial correlation analysis of bilateral spinothalamic tract diffusion metrics with clinical indicators

Based on the statistical results of comparisons of quantified parameters of white matter fiber tract of the bilateral STT between the CNSP and HCs groups, the following were included in the correlation analysis: FA values of the left STT (C2 segment), left STT (C5 segment), right STT (C1 segment) and right STT (C2 segment). MD values of the left STT (C1 segment), left STT (C2 segment), left STT (C5 segment), right STT (C1 segment) and right STT (C5 segment).

**Figure 4:**
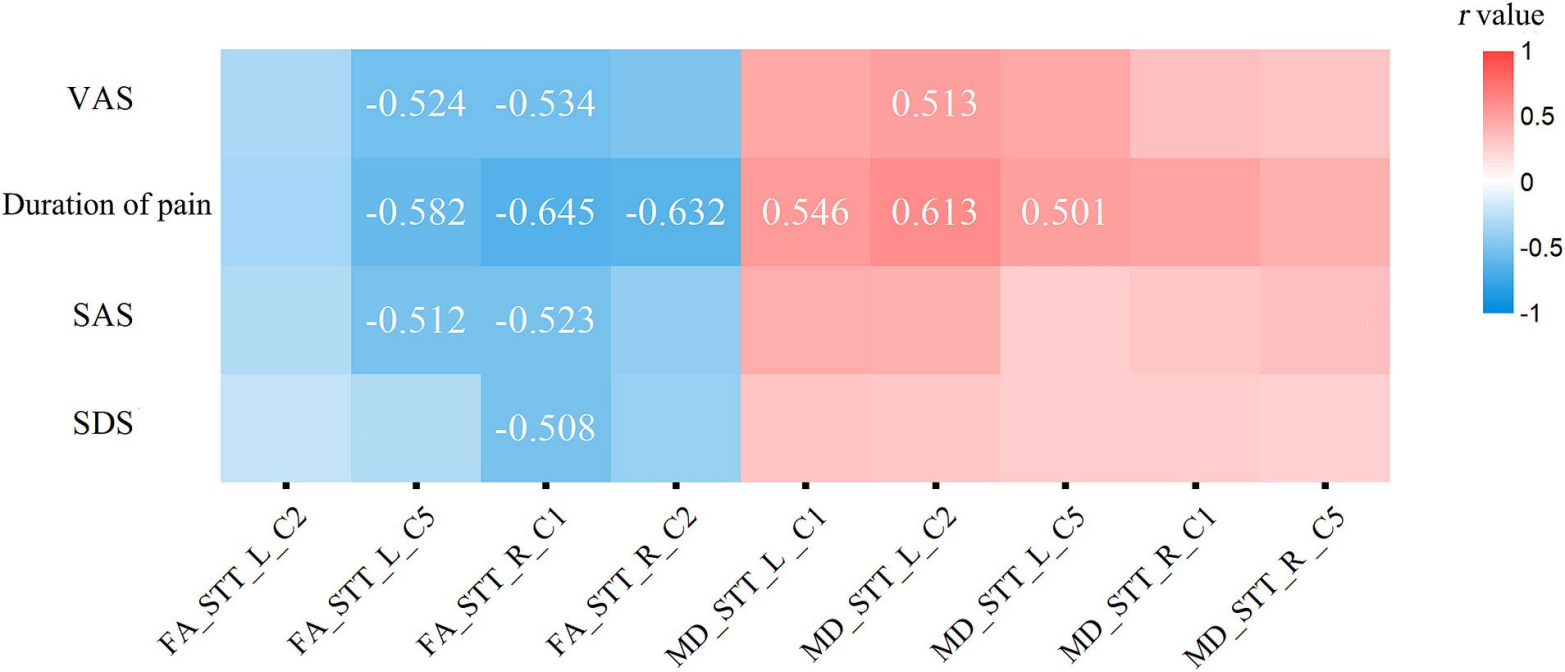
Partial correlation analysis of spinothalamic tract diffusion metrics with clinical measures. Only correlation coefficients (*r*) with statistical significance (Bonferroni *p* < 0.05) are marked. VAS, visual analog scale; SAS, Self-Rating Anxiety Scale; SDS, Self-Rating Depression Scale; FA, fractional anisotropy; MD, mean diffusivity; STT, spinothalamic tract; L, left; R, right.

The age of the subjects was used as a covariate, and Bonferroni correction was applied for multiple comparisons. The partial correlation analysis results indicate that VAS scores are negatively correlated with FA_STT_L_C5 (*r* = −0.524, Bonferroni *p* = 0.042); VAS scores are negatively correlated with FA_STT_R_C1 (*r* = −0.534, Bonferroni *p* = 0.039); VAS scores are positively correlated with MD_STT_L_C2 (*r* = 0.513, Bonferroni *p* = 0.046); the duration of pain is negatively correlated with FA_STT_L_C5 (*r* = −0.582, Bonferroni *p* = 0.016); the duration of pain is negatively correlated with FA_STT_R_C1 (*r* = −0.645, Bonferroni *p* < 0.001); the duration of pain is negatively correlated with FA_STT_R_C2 (*r* = −0.632, Bonferroni *p* < 0.001); the duration of pain is positively correlated with MD_STT_L_C1 (*r* = 0.546, Bonferroni *p* = 0.025); the duration of pain is positively correlated with MD_STT_L_C2 (*r* = 0.613, Bonferroni *p* = 0.002); the duration of pain is positively correlated with MD_STT_L_C2 again (*r* = 0.501, Bonferroni *p* = 0.048); SAS scores are negatively correlated with FA_STT_L_C5 (*r* = −0.512, Bonferroni *p* = 0.046); SAS scores are negatively correlated with FA_STT_R_C1 (*r* = −0.523, Bonferroni *p* = 0.043); SDS scores are negatively correlated with FA_STT_R_C1 (*r* = −0.508, Bonferroni *p* = 0.047) (see Figure 4). The statistically significant results (Bonferroni *p* < 0.05) are annotated in the figure with the corresponding correlation coefficients *r*. Among these, the strongest correlations for FA and MD values are with the disease duration in CNSP patients, followed by VAS scores.

## 4 Discussion

This study employed combined brain and spinal cord DTI imaging techniques, acquiring dMRI data of the brain and spinal cord sequentially during the same scanning session, to quantitatively analyze the FA and MD values reflecting the microstructural characteristics of the STT white matter between CNSP patients and HCs. Our findings indicate that there are segmental changes in the cervical STT white matter microstructure in CNSP patients, which are associated with pain intensity, duration of pain, anxiety, and depression. The implications and potential mechanisms underlying these observations will be explored in subsequent sections.

In this study, we observed that the average FA values in the bilateral intracranial STT of patients with CNSP were reduced compared to those in HCs, whereas MD values were elevated. However, these differences did not reach statistical significance as indicated by the independent sample *t*-test (*p* > 0.05). In contrast, He et al. (2021), utilizing tractography atlas-based analysis (TABS), detected localized microstructural alterations in the STT of patients with primary dysmenorrhea. The discrepancies between these findings may be attributable to our method of averaging diffusion metrics across entire fiber tracts, potentially masking localized abnormalities in white matter microstructure. Future investigations could benefit from employing a methodological approach akin to TABS to more precisely explore microstructural changes in the bilateral intracranial STT in CNSP patients.

In the analysis of cervical STT in CNSP patients, we employed the probabilistic spinal cord template (Dupont et al., 2017) from the Spinal Cord Toolbox (SCT) to delineate the bilateral STT in patients with CNSP. We segmented these tracts into seven sections based on vertebral anatomy and conducted a segment-by-segment comparison with HCs. The analysis revealed that CNSP patients displayed decreased FA and increased MD across multiple STT segments, indicative of white matter microstructural impairment. Similar findings were reported by Mariano et al. (2021), who demonstrated a correlation between pain scores and FA values in the spinal thalamic tracts of multiple sclerosis patients, suggesting a potential association between chronic pain and white matter damage. The underlying physiological mechanisms of such microstructural damage in the STT of CNSP patients remain to be fully elucidated. Some scholars believe that the continuous stimulation of chronic pain may cause prolonged overactivation of neurons in the STT pathway, leading to structural and functional alterations in both neurons and neuroglial cells, thereby compromising the integrity of the white matter (Gmel et al., 2023). Other researchers suggest that chronic pain may induce neuroplastic alterations such as neuronal reorganization and synaptic remodeling, potentially impacting both the function and structure of the STT and resulting in white matter microstructural damage (May, 2008). Furthermore, the interaction between chronic pain and white matter abnormalities in the conduction pathways may be bidirectional. Chronic pain can lead to microstructural changes in the STT’s white matter, while neuronal damage or dysfunction within the STT could exacerbate the amplification of pain signals (Ji et al., 2014). This amplification may intensify the pain experienced by patients. These observations imply that CNSP may not solely originate from nerve root stimulation or compression but could also be associated with alterations in the central nervous system’s white matter pathways involved in pain processing.

Additionally, our study indicates that impairment within the STT is confined to specific segments; however, the underlying causes of this segmental impairment remain unclear. We hypothesize that damage to the STT at the C5 segment could be associated with the pain sites reported by patients. Typically, pain signals from the neck and shoulder regions enter the spinal cord via nerve roots near the C5 segment (Abbed and Coumans, 2007). This excessive activation might induce structural and functional alterations in neurons and neuroglial cells, leading to microstructural damage within the C5 segment of the STT white matter. From an anatomical and functional perspective, signals from various parts of the body gradually converge along the spinal cord before being transmitted to the brain (Wang et al., 2022). Due to the characteristics of this signal convergence process, the C1 and C2 segments may need to process a larger volume of signals, which results in a relatively higher functional load, potentially making them prone to functional overload. Based on this, we hypothesize that the spinothalamic tract (STT) in these segments may be more susceptible to the effects of repeated high-frequency stimulation(such as those induced by chronic pain), resulting in microstructural damage to the white matter. In contrast, the STTs in the C3 and C4 segments may not yet reach the damage threshold. Similarly, Wen et al. (2014) observed in their study on patients with cervical spondylotic myelopathy that decreases in FA values occurred not only at the primary site of compression but also in the C1 and C2 segments, distant from the primary lesion. This suggests that demyelination and axonal damage in cervical spondylotic myelopathy may impact both the lesion sites and remote areas as the disease progresses, paralleling the findings of our research.

In our study, we observed that the FA values across multiple segments of the cervical STT white matter fibers in patients with CNSP were inversely correlated with VAS scores and duration of pain. Conversely, the MD values demonstrated positive correlations with VAS scores and duration of pain. Among these factors, the duration of CNSP exhibited the strongest association with white matter alterations, highlighting the impact of chronicity on neural changes. This suggests that STT white matter microstructure impairment in CNSP patients is intricately linked to the chronicity of pain, with exacerbation of damage as the pain persists. These neural pathways may be crucial in modulating the perception of pain’s intensity and duration. Similarly, Mariano et al. (2021) reported a positive correlation between pain scores and FA values of the spinothalamic tract in patients with multiple sclerosis. It is postulated that prolonged nociceptive stimulation may induce neuroplastic changes (Latremoliere and Woolf, 2009), termed central sensitization, which involves neuronal and synaptic modifications within the pain transmission pathway. The microstructural alterations in these fiber tracts could mirror the extent of central sensitization, correlating positively with disease progression and pain severity (Woolf and Salter, 2000). Moreover, some researchers propose that damage to the white matter microstructure within these pathways could affect the velocity of nerve impulse transmission, potentially altering the timing and fidelity of pain signal relay. Such changes in nerve conduction speed are hypothesized to be linked to the pain’s intensity and chronicity (Fields, 2004).

Furthermore, chronic pain encompasses more than mere sensory perception; it also engages emotional and cognitive dimensions (Seminowicz et al., 2019). Studies have demonstrated that the dynamic functional connectivity density of the right inferior temporal gyrus in CNSP patients are positively correlated with their SDS scores (Ni et al., 2022). Based on this, we hypothesize that changes in the white matter microstructure of the pain conduction pathways in CNSP patients may also be associated with anxiety and depressive symptoms. Our findings indicate that FA values across multiple segments of the cervical STT white matter fibers in CNSP patients are inversely correlated with SAS and SDS scores, whereas MD values show positive correlations with these scores. Damage to the STT may influence brain regions implicated in emotional and cognitive functions, thus affecting patients’ perceptions and assessments of pain. Apkarian et al. (2005) have demonstrated that in chronic pain patients, the brain areas involved in pain processing extend to those responsible for emotional regulation. This suggests that the experience of chronic pain is influenced not only by neural pathways dedicated to pain transmission but also by regions governing emotional responses.

## 5 Limitations

(1) In this study, when analyzing the bilateral intracranial STT, due to technical limitations, only the average FA and MD values of the entire fiber bundle could be calculated, which might obscure local abnormalities in the STT’s white matter microstructure. Future studies could attempt to use a method similar to tractography atlas-based analysis (TABS) (Liu et al., 2017) to further investigate the local microstructural changes in the bilateral intracranial STT of CNSP patients. (2) When extracting the ascending pain conduction pathway, we utilized VPL and S1 as the two ROIs. However, the fiber tracts passing through these ROIs might include the dorsal column-medial lemniscus (DCML) in addition to the STT (Waxman, 2017). Including non-STT fiber tracts in the diffusion metric statistical analysis of the ascending pain conduction pathway may obscure potential results due to the averaging of the entire fiber bundle. Moreover, the S1 region processes sensory input from all parts of the body. If we could use an ROI that specifically receives sensory information from the neck and shoulder regions, more precise results may be obtained. Future studies could explore the use of more detailed anatomical atlases, such as further subdividing the VPL to identify subregions specifically associated with pain, and further subdividing the S1 region to pinpoint subregions that receive sensory inputs exclusively from the cervical and shoulder regions. (3) Our cross-sectional analysis identified atypical changes in the ascending pain pathways of CNSP patients. However, it remains uncertain whether these abnormalities influence the efficacy of pain management strategies or if the white matter microstructure recovers post pain alleviation. This underscores the necessity for ongoing longitudinal investigations. (4) This study noted that some patients exhibited lateralized pain symptoms (pain localized to one side of the neck and shoulder), yet it is uncertain if such lateralization corresponds to asymmetrical changes in neural conduction pathways. Future research should expand the sample size and employ subgroup analyses to specifically explore lateralized pain phenomena. (5) This study did not perform additional MRI scans on patients with chronic neck and shoulder pain to exclude musculoskeletal causes (e.g., rotator cuff injury) that could lead to chronic neck and shoulder pain. It did not consider that chronic neck and shoulder pain caused by different etiologies might lead to different changes in pain ascending conduction pathways. Future studies should consider conducting more detailed subgroup analyses.

## 6 Conclusion

This research identified that patients with CNSP exhibited reduced FA and increased MD in the bilateral intracranial spinothalamic tracts, although these differences were not statistically significant (*p* > 0.05). Conversely, significant abnormalities were observed in specific segments of the bilateral cervical spinothalamic tracts (*p* < 0.05), correlating with variations in pain intensity, illness duration, and levels of anxiety and depression. These findings contribute a novel neuroimaging perspective to the evaluation and elucidation of the pathophysiological mechanisms underlying chronic pain in the ascending conduction pathways. Additionally, this insight may aid in the development of targeted intervention strategies for the ascending pain pathways.

## Data Availability

The raw data supporting the conclusions of this article will be made available by the authors, without undue reservation.
