# Supplementary material for: Exploratory study on the ascending pain pathway in patients with chronic neck and shoulder pain based on combined brain and spinal cord diffusion tensor imaging
Source: Front Neurosci. 2025 Feb 12;19:1460881. doi: 10.3389/fnins.2025.1460881 (PMC11861079; doi:10.3389/fnins.2025.1460881)
Supplement: Supplementary file 1 [file Data_Sheet_1.docx]

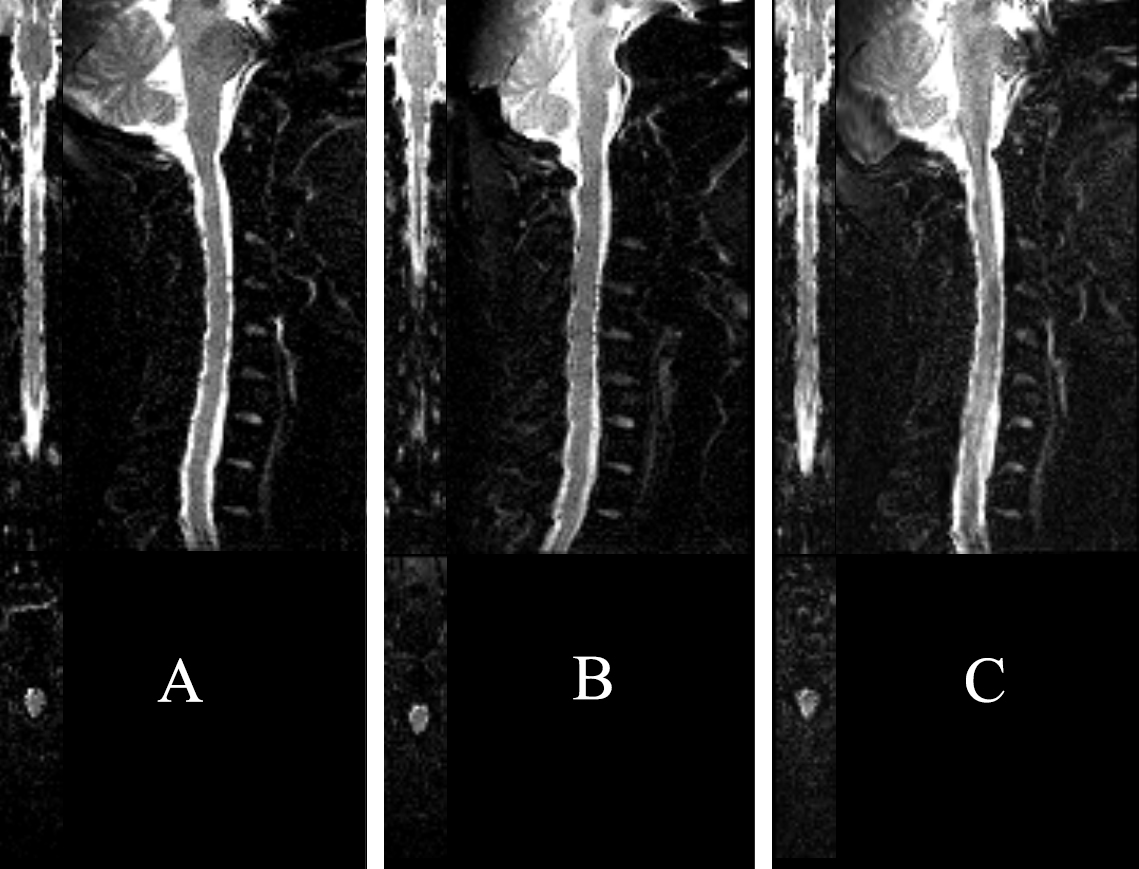


**Figure 1** Figure 1A shows a B0 image in the Anterior-to-Posterior encoding direction from a patient with Chronic Neck and Shoulder Pain, Figure 1B shows the corresponding B0 image in the Posterior-to-Anterior encoding direction, and Figure 1C shows the corresponding B0 image after topup and eddy correction.

**
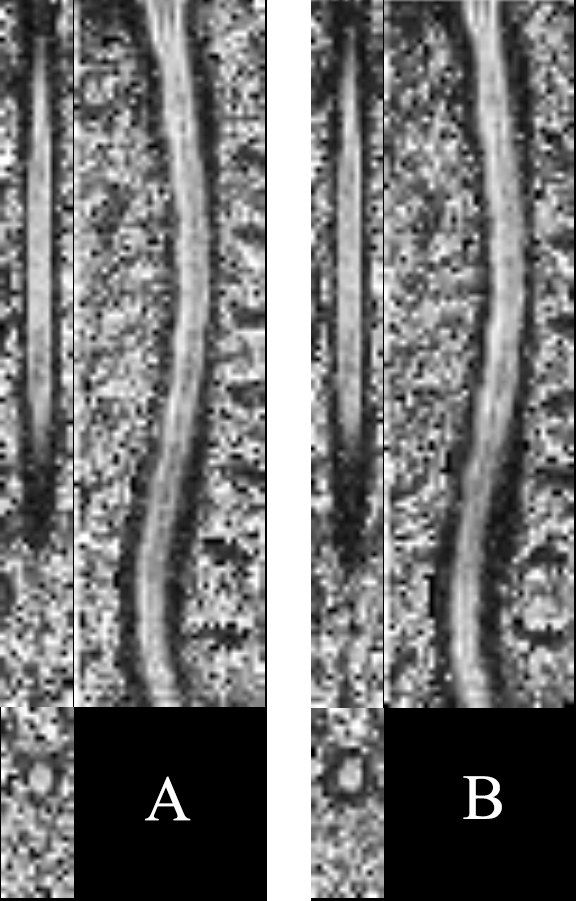
**

**Figure 2** Figure 2A shows the fractional anisotropy image of a Chronic Neck and Shoulder Pain patient before topup and eddy correction, and Figure 2B shows the corresponding fractional anisotropy image after topup and eddy correction.
